# Neck Findings in Hanging: A Cross-Sectional Comparative Study of Conventional Autopsy and Postmortem CT-Based Virtual Autopsy

**DOI:** 10.7759/cureus.92518

**Published:** 2025-09-17

**Authors:** Daunipaia Slong, Prabal Das, Kishanth S, Lokesh Ravi Varma Naidu, Bhaskar Mukherjee, Donboklang Lynser, Amarantha Donna Ropmay, Amar J Patowary

**Affiliations:** 1 Forensic Medicine, North Eastern Indira Gandhi Regional Institute of Health & Medical Sciences, Shillong, IND; 2 Forensic Medicine and Toxicology, All India Institute of Medical Sciences, Rishikesh, Rishikesh, IND; 3 Radiodiagnosis, North Eastern Indira Gandhi Regional Institute of Health & Medical Sciences, Shillong, IND

**Keywords:** hanging, neck findings, postmortem computed tomography, suicide, virtual autopsy

## Abstract

Introduction

Suicide is a significant public health concern, causing numerous deaths each year, with hanging accounting for the majority of these fatalities. In our country, such deaths require a medicolegal autopsy, even though most relatives of the deceased are often reluctant to consent. Therefore, this study was conducted to evaluate the efficacy of virtual autopsy (VA) using postmortem computed tomography (PMCT) in detecting neck findings in hanging, in comparison to conventional autopsy (CA).

Materials and methods

This study included 19 cases of hanging brought to a tertiary care center for medicolegal autopsy. All cases underwent VA using PMCT, followed by CA. Neck findings obtained from both autopsy modalities were subsequently compared.

Results

In this cross-sectional study, 19 cases of hanging were included, comprising 10 females and nine males. There was strong agreement between VA and CA in identifying the ligature material. Examination of the ligature mark showed that VA could detect its presence with 89.5% accuracy and accurately determine the knot position in 84.2% of cases. Fracture of the hyoid bone was observed in one case and was detected by both VA and CA.

Conclusions

VA using PMCT is effective in determining the neck findings in hanging, especially those of the skin and bony structures. Thus, it can be used as a supplement to the CA in hanging. This will help to reduce the total duration of the autopsy while respecting the sentiments of the next of kin by handing over the body of their loved one intact and without deformity.

## Introduction

Suicide is a major global health problem, causing over 700,000 deaths annually and accounting for approximately one in every 100 deaths worldwide in 2019 [1]. In India, the number of suicides has shown an increasing trend over the past five years (2018-2022), reaching over 170,000 deaths in 2022, with a rate of 12.4 per lakh population [2]. Although there are several methods of suicide, hanging is the most commonly adopted means in most countries, including India. According to the National Crime Records Bureau, hanging accounts for more than half (58.2%) of all suicides in India [2].

Death due to hanging necessitates an autopsy, as it is legally required for all unnatural deaths in the country. However, families are often reluctant to consent to an autopsy for various reasons, a phenomenon also reported previously in this region [3]. This has spurred increased interest in less invasive procedures such as virtual autopsy (VA) [4], with the All India Institute of Medical Sciences, New Delhi, being the first institution in India to establish such a center, followed by our institute [5]. Therefore, this study was conducted to evaluate the diagnostic accuracy of VA using postmortem computed tomography (PMCT) in detecting neck findings in hanging cases, compared with conventional autopsy (CA).

## Materials and methods

This cross-sectional study was conducted in the Department of Forensic Medicine at the North Eastern Indira Gandhi Regional Institute of Health & Medical Sciences between January 1, 2023 and December 31, 2024. All cases of alleged hanging brought for autopsy during this period were included. Each case first underwent whole-body PMCT with VA, followed by CA.

Virtual autopsy by PMCT

PMCT was performed with the body in a supine position inside a sealed body bag, using a 32-slice CT scanner (Canon Aquilion Start, Canon Medical Systems Corporation, Ōtawara, Japan) installed in the Department of Forensic Medicine. Scanning parameters were 120 kV and 70 mAs, and the raw data were processed into 1 mm-thick slices. The acquired CT images in Digital Imaging and Communications in Medicine (DICOM) format were transferred to a workstation with Inobtech DICOM Viewer Professional Edition 2.9.0.10076, where the findings were evaluated.

The neck images were examined and interpreted using multiplanar reconstruction and 3D volume-rendering techniques by a radiologist who was aware that the cases involved hanging but did not have access to the CA findings.

CA

CA was performed by one of three autopsy surgeons in the department, following standard autopsy protocols [6]. The procedure included both external and internal examinations, with detailed inspection of the neck structures, including the ligature mark, subcutaneous tissues, neck muscles, and osteocartilaginous structures such as the hyoid, thyroid cartilage, and cervical vertebrae. The autopsy surgeons were blinded to the PMCT findings.

Statistical analysis

Findings from both CA and PMCT were collected and entered into IBM SPSS Statistics for Windows, Version 31.0 (Released 2025; IBM Corp., Armonk, NY, USA), for analysis. Cohen’s kappa coefficient was calculated to assess agreement between the two modalities. Interpretation of kappa values was based on the criteria established by Landis and Koch [7].

## Results

During the study period, a total of 137 medicolegal autopsies were conducted, of which 19 were cases of hanging and were included in this study. The decedents had a mean age of 28.89 years (SD = 13.58), ranging from 12 to 55 years, and comprised nine males (47.4%) and 10 females (52.6%).

**Figure 1:**
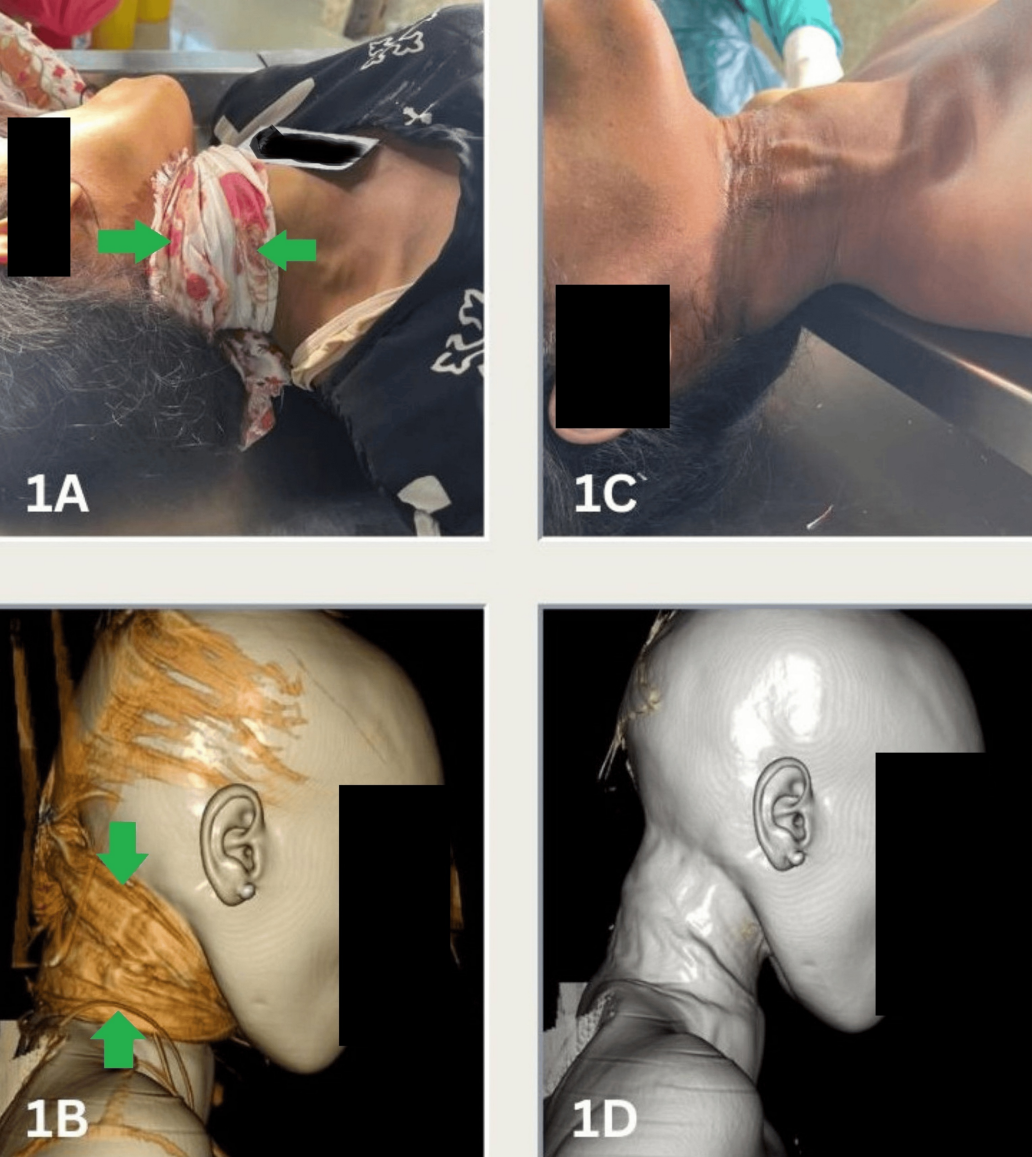
Photograph and 3D volume-rendered image of a ligature (green arrows) passing twice around the neck (1A, 1B) and the corresponding ligature mark on the neck (1C, 1D)

A review of police reports and information provided by relatives indicated that a variety of materials, including nylon rope, bedsheets, and curtains, were used as ligatures for hanging (Table 1). Among the cases, the ligature was present on the body at the time of autopsy in eight cases and absent in 11 cases. In all cases where the ligature material was present, PMCT successfully identified it (Table 2). However, the texture and pattern of the ligature could not be fully ascertained on PMCT in cases involving soft materials, although the pattern was partially discernible when hard materials such as nylon rope were used. The type of knot and multiplicity of the noose around the neck (Figure 1) were assessable on PMCT in all 8 cases and demonstrated perfect agreement with CA findings (κ = 1).

**Table 1:** Details of hanging

Characteristics	Findings	Frequency
Place of occurrence	Indoor	14
	Outdoor	5
Type of hanging	Complete	12
	Partial	7
Ligature material	Rope	7
	Cotton cloth	4
	Bedsheet	3
	Gamocha	2
	Curtain	1
	Sling bag	1
	Bamboo cord	1

**Table 2:** Comparison of the characteristics of ligature material by CA and VA CA, conventional autopsy; VA, virtual autopsy

Characteristics	Findings	CA	VA	Statistics
Type of knot	Slipknot	3	3	κ = 1
	Fixed knot	5	5	
Number of turns	Single	7	7	
	Double	1	1	

Examination of the ligature mark (Table 3) using 3D volume-rendering techniques revealed that PMCT detected the mark with a high level of accuracy (89.5%), identifying 17 of 19 cases when compared with CA (19/19). In two of these 17 cases, the mark was faintly visible during CA, making it difficult to detect on PMCT, where it appeared only as a superficial groove on the front and side of the neck. In the remaining 15 cases, the mark appeared as a well-defined groove on both modalities.

**Table 3:** Comparison of the characteristics of ligature marks by CA and VA CA, conventional autopsy; VA, virtual autopsy

Characteristics	Findings	CA	VA	Statistics
Detection	Detected	19	17	Accuracy = 89.5%
	Not detected	0	2	
Continuity	Incomplete	17	13	κ = 0.433
	Complete	2	3	
	Not detected	0	3	
Level in relation to the thyroid	Above	16	14	κ = 0.555
	At	3	3	
	Not detected	0	2	

Further examination by CA revealed that the ligature mark was incomplete in 17 cases and complete in two cases. When compared with PMCT, agreement between the two modalities was observed in 15 cases (13 incomplete and two complete), with disagreement in one case (incomplete by CA, complete by PMCT). In three cases, PMCT failed to detect the discontinuity of the ligature mark, corresponding to the two cases in which it failed to detect the mark itself and one of the cases with faint marks mentioned above. Overall, moderate agreement was found between the two modalities (κ = 0.433), which was statistically significant (p = 0.002). Similarly, PMCT identified the knot position with 84.2% accuracy compared with CA (19/19) but failed to detect the suspension point in the same three cases.

While comparing the relationship of the ligature mark with the thyroid cartilage (Table 3), agreement between the two autopsy modalities was observed in 16 cases (14 cases above the thyroid cartilage and two cases at the level of the thyroid cartilage), with disagreement in one case (above the thyroid by CA and at the level of the thyroid by PMCT). This demonstrates a moderate level of agreement between the two modalities (κ = 0.555), which was statistically significant (p = 0.002). However, PMCT failed to detect this finding in two cases because the ligature mark itself was not visualized.

**Figure 2:**
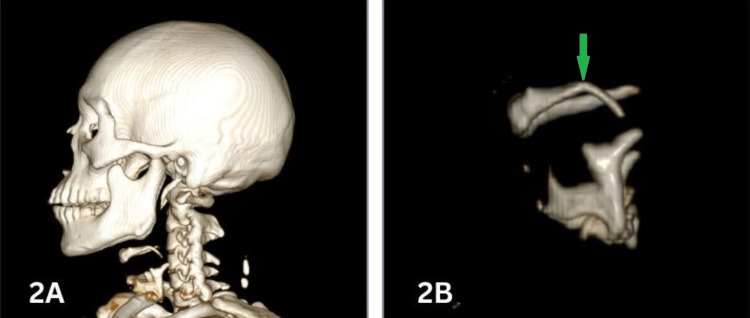
3D volume-rendered images showing the fracture of the left greater cornu of the hyoid bone (green arrow), with 2A displaying the fracture alongside other bony neck structures and 2B showing the fracture after bone segmentation

Examination of the bony structures of the neck revealed a fracture in only one case, involving the left cornu of the hyoid bone, which was identified by both modalities (Figure 2). Notably, soft tissue and neck muscle hemorrhages were absent in all cases.

## Discussion

Wherever a ligature is present, it should be carefully examined to assess its correlation with the ligature mark on the neck, the type of knot applied, and its position on the neck [8]. In cases of hanging, easily accessible materials such as ropes, bedsheets, and similar items are commonly used as ligatures. This trend is observed in our study as well as reported by other authors [9]. When comparing the ability of CA and PMCT-based VA to detect the ligature, perfect agreement (κ = 1) was observed, consistent with findings reported by Lyness et al. [10]. Detection becomes more challenging when the ligature is thin, flat, and tightly applied to the skin, although its presence can still be identified by PMCT. The ligature pattern can be assessed by PMCT, particularly when a rope is used, but broad, soft ligature materials limit the ability to specify the type. Previous studies indicate that standard axial images and 3D volume rendering techniques can usually identify the ligature type [10]. Other features, such as the number of turns around the neck and the knot details, were also evaluated, showing perfect agreement (κ = 1) between the two modalities, corroborating earlier findings [10].

**Figure 3:**
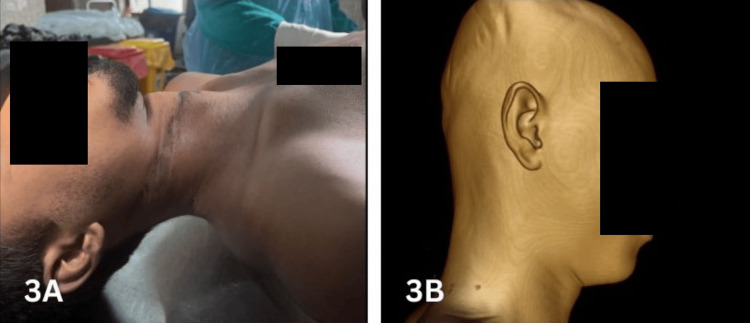
Photograph (3A) and 3D volume-rendered image (3B) showing a superficial ligature mark on the neck, difficult to visualize during VA VA, virtual autopsy

**Figure 4:**
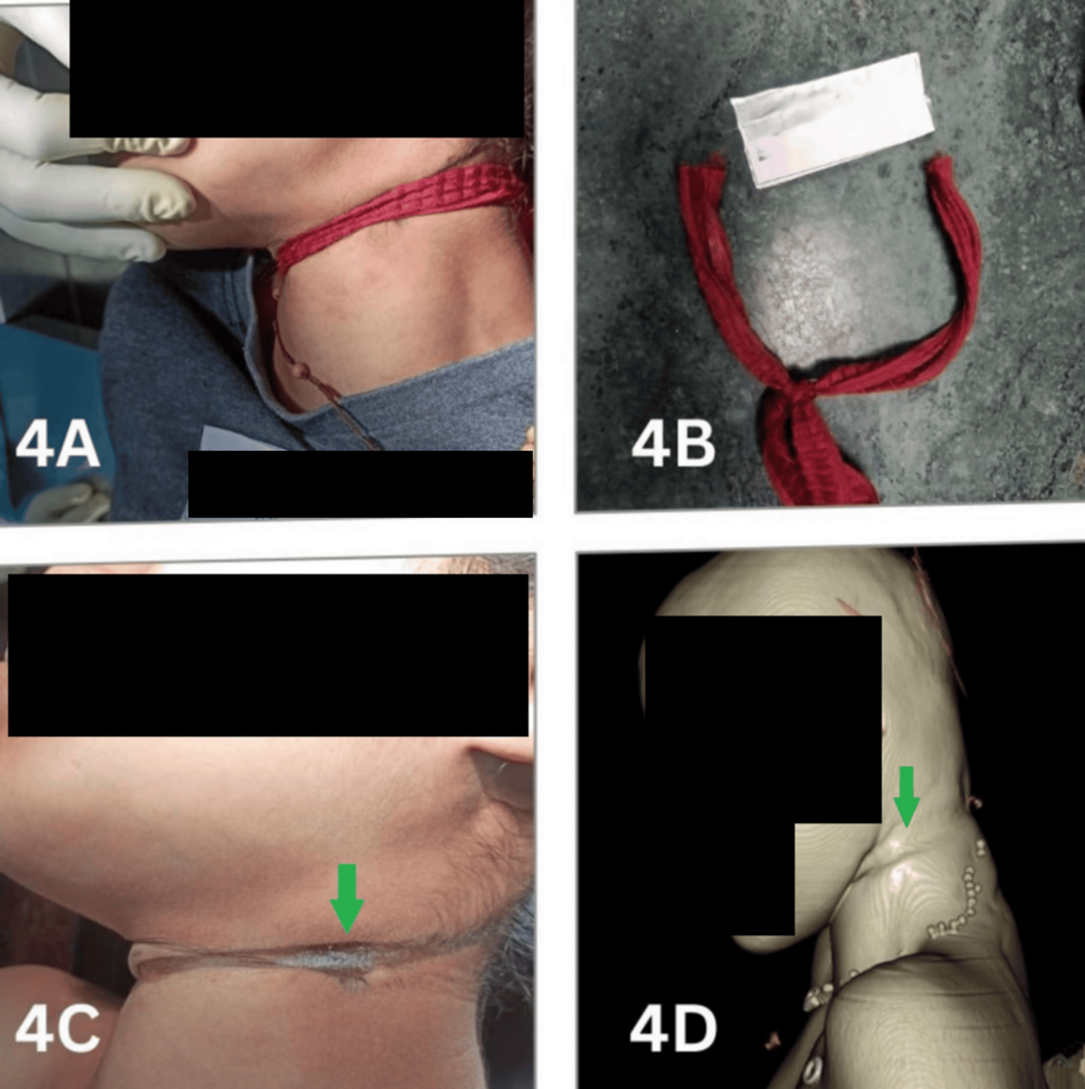
Photograph of a soft, thin ligature (4A, 4B) and the corresponding well-defined ligature mark (green arrow) on the neck as shown in the photograph (4C) and 3D volume-rendered image (4D)

Autopsy of suspected hanging cases requires a meticulous examination of the ligature mark on the neck, which is a vital finding. Typically, the ligature mark is described according to its length, width, depth, pattern, color, direction, completeness, and location relative to the thyroid cartilage [8]. It appears as a groove or furrow, with its prominence influenced by the duration of suspension, type of ligature, and height of suspension [11]. In our study, PMCT identified the ligature mark with 89.5% accuracy, with better visualization when a rope or other hard object was used compared to soft ligature materials. In the two cases where PMCT failed to detect the mark, it was superficial and faint (Figure 3), corresponding to soft ligature materials. Similarly, Khan et al. [12] reported a sensitivity of 92% (CI = 80.77-97.78%) for PMCT in detecting ligature marks using 3D reconstruction techniques. However, other studies report minimal agreement (κ = 0.223) between CA and PMCT for ligature mark identification, likely due to challenges posed by skin folds, partial visibility, or shallow/faint marks with soft ligatures [10,12]. In one case (Figure 4), a soft, thin ligature produced a well-defined, deep groove, likely due to prolonged suspension and tight application. Broad and soft ligatures may also create multiple congested areas, which appear on PMCT as raised areas or ridges along the ligature mark [8].

Regarding the suspension point, a study on 50 hanging cases reported that PMCT identified it in 43 cases compared with 48 cases by CA, demonstrating strong agreement (κ = 0.832) [10]. In our study, PMCT correctly identified the knot position in 84.2% of cases, with failures corresponding to the two cases in which the ligature mark was not visualized by PMCT and another case with a faint mark. This highlights that a detailed assessment of faint ligature marks, including width, direction, and suspension point, is better achieved with CA, even if PMCT detects the presence of the mark. Consistent with the literature, the ligature mark in hanging is most often located above the thyroid cartilage [13]. Comparing PMCT with CA for identifying the level of the ligature mark relative to the thyroid cartilage showed moderate agreement (κ = 0.555).

Fracture of the hyoid bone is rare in individuals under 40 years of age [11,12]. The efficacy of PMCT in detecting fractures is well documented [12], with studies showing strong agreement between PMCT and CA [14]. PMCT effectively detects fractures of the bony neck structures, with some studies reporting 100% sensitivity and specificity for thyroid and hyoid fractures [12]. A systematic review found an overall sensitivity of 0.70 and specificity of 0.92 for hyoid fractures and a sensitivity of 0.8 and specificity of 0.76 for thyroid cartilage fractures [15]. While some authors report equivalence between PMCT and autopsy for detecting laryngeal fractures [14], higher-resolution scans can detect more subtle fractures [16]. Conversely, Lyness et al. [10] found weak to moderate agreement for thyroid (κ = 0.538) and hyoid fractures (κ = 0.555). In our study, one fracture was observed in a 40-year-old male, involving the left cornu of the hyoid bone, detected by both modalities.

Soft tissue hemorrhages were absent in our cases and are known to be difficult to identify using PMCT [12]. Gascho et al. [17] reported that autopsy detected three times more soft tissue hemorrhages than PMCT. PMCT angiography has been suggested to overcome this limitation, allowing detection of vascular injuries and hematomas missed on standard PMCT [18]. MRI is another reliable modality to improve the detection of neck tissue hemorrhages [17].

## Conclusions

VA is a relatively new technique in the field of forensic medicine. Although CA remains the gold standard, VA can often supplement it and, in some cases, may even replace CA with comparable or superior accuracy. It is particularly effective in identifying skin and bony findings of the neck in hanging cases. Therefore, it can be employed either as a supplement to CA or in combination with external examination and PMCT-based VA, without the need for full body dissection, in selected cases of hanging. Furthermore, because VA requires significantly less time than CA, it may be considered a viable alternative in such cases.
